# Patient-provider communication about gestational weight gain among nulliparous women: a qualitative study of the views of obstetricians and first-time pregnant women

**DOI:** 10.1186/1471-2393-13-231

**Published:** 2013-12-11

**Authors:** Elizabeth A Duthie, Elaine M Drew, Kathryn E Flynn

**Affiliations:** 1 Center for Patient Care and Outcomes Research, Medical College of Wisconsin, 8701 Watertown Plank Rd., Milwaukee, WI, USA; 2 Department of Family and Community Medicine, Medical College of Wisconsin, 8701 Watertown Plank Rd., Milwaukee, WI, USA

**Keywords:** Pregnancy, Gestational weight gain, Patient-provider communication, Prenatal care, Obstetrics, Qualitative research

## Abstract

**Background:**

In 2009 the Institute of Medicine updated its guidelines for weight gain during pregnancy, in part because women of childbearing age now weigh more pre-pregnancy and tend to gain more weight during pregnancy than women did when the previous set of guidelines were released in 1990. Women who begin pregnancy overweight or obese and women who gain weight outside IOM recommendations are at risk for poor maternal and fetal health outcomes. With these concerns in mind, we examined what obstetricians communicate about gestational weight gain to their pregnant patients and how nulliparous patients perceive weight-related counseling from their obstetricians.

**Methods:**

We conducted one-on-one, semi-structured interviews with 19 nulliparous women and 7 obstetricians recruited from a single clinic at a large academic medical center in the United States. Interviews were transcribed verbatim and analyzed inductively using thematic analysis.

**Results:**

We identified 4 major themes: 1) Discussions about the amount and pace of gestational weight gain: obstetricians reported variation in the frequency and timing of weight-related discussions with patients while most patients said that weight was not emphasized by their obstetricians; 2) The content of communication about nutrition and physical activity: obstetricians said they discuss nutrition and activity with all patients while most patients reported that their obstetrician either discussed these topics in general terms or not at all; 3) Communication about postpartum weight loss: obstetricians said that they do not typically address postpartum weight loss with patients during prenatal visits while patients had concerns about postpartum weight; and 4) Patient feelings about obstetrician advice: most patients said that their obstetrician does not tend to offer “unsolicited advice”, instead offering information in response to patient questions or concerns. Women were divided about whether they desired more advice from their obstetrician on weight gain, nutrition, and activity.

**Conclusions:**

Our analysis revealed discrepancies between obstetricians’ and patients’ perceptions of their weight-related clinical interactions. Our findings suggest that there is a missed opportunity to use prenatal visits as opportunities to discuss healthy eating and exercise during pregnancy, the postpartum period, and beyond. Additional research on the design, implementation, and testing of interventions to address prenatal nutrition and physical activity is warranted.

## Background

Obesity is among the most significant health issues of the 21^st^ century [1-3]. Weight gain has unique significance for pregnant women and their health care providers, as pregnancy is a rare occasion when weight gain is medically prescribed [4]. Gestational weight-gain (GWG) warrants attention because both inadequate and excessive weight gain are associated with negative birth outcomes [5,6]. Women who gain weight outside of the recommendations are at risk for gestational diabetes [7], hypertension [8], pre-eclampsia [9], labor and delivery complications [5] including need for cesarean section [10], and postpartum weight retention [5,9]. Infants are also affected. Inadequate and excessive GWG are associated with small-for-gestational age and large-for-gestational age infants, respectively [11,12], hypoglycemia in infants [13], and infant mortality [5,14].

The Institute of Medicine (IOM) in the United States of America provides guidelines for healthcare providers to use when counseling pregnant women on nutrition and physical activity to promote healthy weight gain (Table 1) [5]. Experts recommend that women should be encouraged to engage in at least 30 minutes of moderate intensity exercise most days of the week [5,15,16]. They should not increase their caloric intake during the first trimester of pregnancy, but during the second and third trimesters women require approximately 300 and up to 450 additional calories per day, respectively [17]. While these additional energy requirements are not substantive enough to necessitate literally eating for two, pregnancy does require some dietary adjustment for most women [18], some of whom seek guidance from obstetric care providers [19].

**Table 1:** 2009 Institute of Medicine recommendations for weight gain during pregnancy

**Pre-pregnancy BMI**	**Total weight gain:**		**Rate of weight gain, 2**^**nd **^**and 3**^**rd **^**trimester**	
	**Range in kg**	**Range in lbs**	**Mean (Range) kg/week**	**Mean (Range) lbs/week**
Underweight (< 18.5 kg/m^2^)	12.5-18	28-40	0.51 (0.44-0.58)	1 (1–1.3)
Normal weight (18.5-24.9 kg/m^2^)	11.5-16	25-35	0.42 (0.35-0.50)	1 (0.8-1)
Overweight (25.0-29.9 kg/m^2^)	7-11.5	15-25	0.28 (0.23-0.33)	0.6 (0.5-0.7)
Obese (≥ 30.0 kg/m^2^)	5-9	11-20	0.22 (0.17-0.27)	0.5 (0.4-0.6)

Source: Institute of Medicine and National Research Council of the National Academies [5].

Little is known about how these guidelines affect the ways obstetricians counsel patients about weight gain, and the ways patients perceive that counseling. In one study, nearly 27 percent of pregnant women reported that they received no GWG advice at all from their obstetrician, and 36 percent reported that they received advice to gain weight outside of the IOM’s recommendations [20]. In a Canadian study, over half of women reported that no discussions about GWG took place with their health care provider [21], while another Canadian-based survey found that 95% of health care providers reported that they did counsel pregnant women on appropriate GWG [22]. Qualitative research on the meaning of quality in prenatal care, however, has shown that Canadian obstetric care providers and patients agree nutrition and weight are important topics for prenatal counseling [23]. Another study showed that more than 20 percent of pregnant women had GWG goals that were outside the IOM’s recommendations [24]. In postpartum interviews, overweight and obese women reported that their providers counseled them to gain more weight than guidelines advise or did not provide guidance at all [25]. Surveys of obstetric care providers have found that few comply fully with best-practice recommendations [26], and focus groups [27] and interviews [28,29] with prenatal care providers identified several perceived barriers to discussing GWG. Interviews with Australian midwives showed that weight gain was not perceived as a priority and that midwives were concerned about the psychological effects discussing weight might have on their patients [30]. American midwives and physicians have also reported that compared to competing issues, such as smoking, substance abuse, and domestic abuse, weight is not a priority in prenatal care [29].

Patient-provider communication is a key aspect of the doctor-patient relationship [31,32]. Communication specifically about GWG remains understudied, as few studies have elicited the perspectives of both patients and providers [33,34]. In previous work in Japan, Haruna et al. [33] conducted focus groups with obstetric care providers and with pregnant women. They found that respondents in both groups generally dismissed medical guidelines and agreed that pregnancy weight gain should be kept to a minimum. In England, Olander et al. [34] conducted a similarly designed focus group study and found that pregnant women reported being unconcerned about GWG, and their providers reported uncertainty about what kind of GWG counseling to offer. Another British study that focused on obese pregnant women found that they reported being cognizant of their weight status and its implications for their pregnancy but felt judged and criticized when providers raised the issue with any frequency [35]. Midwives in this same study reported feeling their weight-related counseling was constrained by societal norms that make commenting on a person’s weight taboo. No studies of this type have been conducted in the United States.

In this paper, we describe the perspectives of pregnant women and their obstetricians regarding their discussions about GWG during routine prenatal clinic visits. Specifically, we address two aims: (1) to describe what obstetricians communicate about GWG to their patients, as well as what they communicate to their patients about weight loss in the postpartum period; (2) to describe the experiences women have communicating with their obstetricians about GWG.

## Methods

### Participants

We recruited physicians who were seeing obstetric patients in the obstetrics and gynecology clinic at a large academic medical center in the Midwestern United States with an email inviting them to participate in an interview about recommendations for GWG. We recruited a convenience sample of patients from the same clinic using flyers posted in the clinic and through referrals from clinic staff. Eligible patients were 18 years of age or older and between 29 and 40 weeks gestation at the time of the interview. We conducted interviews in the third trimester because we wanted to talk to women after they had experienced significant bodily changes and had established a relationship with their provider.

### Data collection

A trained female member of the research team (EAD) conducted interviews with patients and physicians between June 2011 and January 2012, using a semi-structured interview guide (example questions and responses are provided in Tables 2 and 3). A multidisciplinary committee of researchers and clinicians including social and clinical psychologists, a medical anthropologist, and an obstetrician reviewed the interview guides. Participants also completed a short survey to collect basic demographic information to allow us to situate their responses in a demographic context. Interviews were audio-recorded and transcribed. Written transcripts were managed using ATLAS.ti version 6.2. Patients were compensated with a $30 gift card for their time. The study protocol was approved by the institutional review board at the Medical College of Wisconsin and Froedtert Hospital. All participants provided written informed consent.

**Table 2:** Interview questions and example responses by content area: patients

**Content area**	**Question(s)**	**Example patient responses**
Communication about gestational weight gain	What has your doctor told you about gaining weight during pregnancy?	The only thing I’ve heard [about my weight gain] is that it’s been steady. It sounds like that is maybe a good thing, I wasn’t really sure…I’m curious nowadays if they just don’t talk about it as much, or that they are afraid of offending people, or if it’s political correctness, ‘cause I haven’t heard much from them [about weight]. –Pre-pregnancy BMI 25.1, 14.1 kg (31 pounds) gained at 32.5 weeks
	What do you think of your doctor’s advice?	
The content of advice: nutrition and physical activity	How has advice from your doctor changed your eating habits?	I am measuring small, and I haven’t gained the most I should gain, so he said that for me it’s fine, go ahead, and encouraging me to make sure I feel free to eat. Because I’m small, I’m guessing they might worry that I don’t eat like I should. I’ve never been criticized for it. He’s never said, ‘oh, you’re not eating enough.’ He just encourages me, ‘Feel free to eat the ice cream, and whatever you want.’ –Pre-pregnancy BMI 18.2, 11.8 kg (26 pounds) gained at 37 weeks
	How has advice from your doctor changed your exercise habits?	
Postpartum weight management	What has your doctor told you about losing weight after your baby is born?	She hasn’t said anything. Not that I can remember. –Pre-pregnancy BMI 23.7, 22.7 kg (50 pounds) gained in 37 weeks
		I’ve done a lot of research on my own, but I haven’t heard anything from the doctor. –Pre-pregnancy BMI 25.1, 14.1 kg (31 pounds) gained at 32.5 weeks
Feelings about obstetrician advice	What do you think of your doctor’s advice?	Overall, I'd want to hear a little bit more from [my obstetrician], 'cause I don't know what to ask sometimes. –Pre-pregnancy BMI 23.3, 12.7 kg (28 pounds) gained at 40 weeks
	What would you like to know about pregnancy weight gain and postpartum weight loss from your doctor?	[I hope my obstetrician will] let me know when it's okay to start working out and how much I can do. And a healthy diet program where I can still get the baby everything he needs through the breast milk, and still be healthy myself. –Pre-pregnancy BMI 35.8, 14.1 kg (31 pounds) gained at 35.5 weeks

**Table 3:** Interview questions and example responses by content area: obstetricians

**Content area**	**Question**	**Example obstetrician responses**
Communication about gestational weight gain	What is your philosophy about discussing pregnancy weight gain with your patients?	I talk to all my patients about what their starting BMI is, and what their expected weight gain will be during the pregnancy…And I let them know that I will be checking in on their weight at every visit.—Female
		I will usually not talk about it at the first visit ‘cause there’s a lot of other things at that point, and frequently nausea is still an issue and worrying about how much they will gain is less important than keeping down what they are currently eating. Usually, slightly before or at the midway point I’ll talk about that, and just tell them what I think they’re on a rate to gain, as an estimate, and whether I think that’s good or not.—Male	
The content of advice: nutrition and physical activity	What do you advise your patients about nutrition, physical activity, and weight gain?	I think exercise is really important, and gets put to the side. A lot of people have the perception that you can’t exercise during pregnancy, so just educating that you can and should continue exercise…I feel like people have a little bit more leeway or latitude with what they eat if they’re consistently active. And so I keep stressing that.—Female	
		Those that are sedentary at the beginning of pregnancy, I generally recommend they start walking, or something that’s equally low impact, at least three days a week for 30 minutes.—Male	
Postpartum weight management	What do you tell your patients about weight loss in the postpartum period?	[I] generally advise women that very few women are back to their pre-pregnancy weight at the time of the six-week visit, but that…starting to increase their activity…or getting back into an exercise routine, is really the best way.—Female	
		I usually don’t talk about this until the six-week postpartum checkup… Many of my patients actually by six weeks are back to their normal weight. Those that aren’t, I don’t think I ever give them a hard time about it, but we do try to talk about diet and exercise at that visit.—Male	

### Analysis of interview data

We conducted a thematic analysis of interview transcripts in two stages, corresponding with the inductive approaches of open coding and axial coding [36]. For the open coding, two coders (EAD and EMD) separately conducted line-by-line coding of a sample of transcripts to create codebooks. The codebooks were then refined into a master codebook through comparison and categorization, with discrepancies resolved through discussion on the interpretation of the codes and their properties and dimensions [37]. Axial coding followed the open coding, wherein we reviewed the transcripts and highlighted references to each of the elements identified in the codebook [36]. The identification of these elements (or domain analysis) allowed us to take the next step of extracting and sorting the segments of text or “themes” that would elucidate the full range of participant perceptions of pregnancy weight gain [36]. We coded and analyzed obstetrician and patient transcripts separately.

## Results

Nineteen patients and seven obstetricians completed interviews. Patient characteristics are presented in Table 4. Patients were diverse with respect to age and race. Patients were nulliparous; two patients had previously experienced miscarriages earlier than 12 weeks gestation and had not experienced GWG prior to their current pregnancy. With the exception of one patient who was carrying twins, patients had singleton pregnancies. Physicians were diverse with respect to sex (3 men, 4 women), years since graduation from medical school (1985-2005), and years on the faculty at the clinic (2–20).

**Table 4:** Patient characteristics

**Characteristic**	**Pre-pregnancy BMI < 25.0**	**Pre-pregnancy BMI ≥ 25.0**	**Total**
	**N = 8**	**N = 11**	**N = 19**
	**% (N) or Mean (Range)**	**% (N) or Mean (Range)**	**% (N) or Mean (Range)**
Race/Ethnicity			
American Indian/Alaska Native	0 (0)	9% (1)	5% (1)
Black/African American	0 (0)	9% (1)	5% (1)
Hispanic/Latino	13% (1)	9% (1)	11% (2)
Indian	13% (1)	9% (1)	11% (2)
Multiple	13% (1)	9% (1)	11% (2)
White	62% (5)	55% (6)	58% (11)
Education			
In high school	0 (0)	9% (1)	5% (1)
High school	13% (1)	9% (1)	11% (2)
Some college	13% (1)	9% (1)	11% (2)
Bachelor’s	25% (2)	46% (5)	37% (7)
Post-baccalaureate	50% (4)	27% (3)	37% (7)
Age	29.9 (22–36)	29.4 (20–40)	28 (20–40)
Starting BMI	22.7 (18.2-23.7)	30.4 (25.1-43.3)	27.2 (18.2-43.3)
Weight gained: Kilograms	12.7 (7.7-22.7)	13.7 (3.2-27.2)	13.2 (3.2-27.2)
Pounds	27.9 (17–50)	30.1 (7–60)	29.1 (7–60)
Gestation in weeks	34.6 (29–40)	35.9 (33–40)	35.4 (29–40)

Analysis of transcripts revealed four prominent themes related to patient-provider communication about GWG: (1) communication about the amount and pace of GWG; (2) the content of nutrition and physical activity communication; (3) communication about postpartum weight loss; and (4) patients’ feelings about their provider’s weight-related counseling.

As previously described, we coded and analyzed obstetrician and patient transcripts separately. In what follows, we report them together (interspersed) by theme, in order to make clear the similarities and differences in the perspectives of the two groups.

### Communication about gestational weight gain

Obstetricians generally preferred that their patients gain weight within the guidelines set by the IOM but reported differences in the frequency and timing of their weight-related discussions. Some said they addressed the topic with patients early in the pregnancy so patients would know upfront what to expect, while others reported that they intentionally did not discuss weight at the first few prenatal visits because other topics are more important. One obstetrician said that she discussed weight with every patient at every prenatal visit.

Patients reported that weight did not seem like a high priority for their obstetricians during prenatal visits. Most women said that their doctors talked to them early in their pregnancy, most often at their first appointment, about how much weight they should expect to gain, but that weight was not a focus of subsequent appointments. A few patients speculated that doctors might avoid the topic because they assume it is uncomfortable for women, or that doctors would address weight gain if it became a serious problem.

*[There was] not much conversation nutrition-wise, weight-wise. And by not much, I guess not any…Maybe it’s because… since I carry the weight well, apparently, I don’t look as heavy as I am, even though he’s got it in the charts. And they weigh me at every visit…[Maybe he hasn’t brought it up] because I haven’t gained too much and I haven’t put on the 40 pounds, or the 60 pounds, or some of these stories that you hear, and I wasn’t morbidly obese.* –Patient, pre-pregnancy Body Mass Index (BMI) 30.1, 6.8 kg (15 pounds) gained at 35 weeks

Obstetricians appeared to take patient characteristics into account when they communicated with patients. Obstetricians reported that they might not focus on weight with every patient because they tailor their counseling depending on several factors, including pre-pregnancy BMI (advising women with higher BMIs to gain less weight than women with lower BMIs), the amount of weight the woman has gained, and patients’ anxiety about weight gain. For example:

*If she’s neurotic about it and we’re talking about it every visit because she’s driving that, it doesn’t matter [whether I think we need to discuss it], I’m still gonna have to talk about that. I’ve still gotta talk her off the ledge each time, whether it’s appropriate or inappropriate or whatever it is, we gotta have the conversation.* –Obstetrician, Male

When a patient has gained weight within expectations, they do not mention GWG. When weight gain is outside expectations, most doctors said they would open a dialogue with the patient about her nutrition and physical activity. If this conversation satisfies the doctor that the woman’s habits are healthy, most doctors will reassure the patient that her weight gain is fine. If this conversation does not, then doctors described taking several different approaches with their patients.

One approach obstetricians described is to offer additional counseling by reviewing what the woman is eating and then identifying areas for improvement. In addition to this counseling, several doctors discussed methods they use to persuade women to moderate their weight gain. One means of persuasion is discussing the complications of pregnancy and childbirth that can arise with excessive weight gain.

*The most effective way I know to help them change things is to let them know the baby could be really big. And if the baby gets bigger there’s an increased risk the baby won’t fit so they would need a caesarean, and depending on their response to that we will go on to the next step, whether it’s an increased risk of the shoulder being trapped and the baby, tiny chance, but real chance, of lifelong disability from it. So I try to slowly ratchet it up if they’re clearly not following a good eating plan.* –Obstetrician, Male

A second approach obstetricians used to persuade women to moderate their weight gain was to focus on the difficulties of postpartum weight loss for women with high GWG.

*I do tell them that if they gain in the recommended range they’re most likely to get back to their pre-pregnancy weight. So, I use it kind of as a way to encourage women to not over-gain during a pregnancy*. –Obstetrician, Female

Patients’ accounts of these encounters were somewhat different. Nearly all women, even those who had gained weight outside the IOM recommendations, said that their doctor had little if anything to say about their weight. When conversations about the amount or pace of weight gain did occur, patients reported that they had prompted the discussion, with doctors rarely raising the subject. Patients said that in these conversations, their doctors reassured them that their weight gain was appropriate. Most women said they were comfortable raising the topic of weight with their doctors, but a few were hesitant about initiating conversations about certain weight-related topics, such as disordered eating habits.

### The content of advice: nutrition and physical activity

Six of the seven physicians reported that they talk about nutrition with every patient at some point during her pregnancy, generally encouraging women to consume a “healthy diet”.

*I tell women to stay away from the fatty foods, sugar foods, just the junk food type things. Just to try to eat healthy, but not to worry about the weight gain…there’s myths that you’re going to have all these cravings for ice cream, craving for all this bad food and it’s all fine to eat all this bad food that you never would eat normally. So, [I provide] more counseling that you should still eat a healthy diet.* –Obstetrician, Female

In contrast, patients reported that their doctors either offered general advice about eating a “balanced diet” or did not discuss nutrition at all. A few women mentioned handouts about nutrition that they received from the clinic but added that their physicians had not reviewed the material with them. Several expressed surprise that they did not receive counseling about foods that pregnant women should avoid, though most women said they looked into this information on their own.

Five of the seven obstetricians reported that they counsel pregnant women on exercise, but less so than on nutrition. Those that discussed exercise emphasized that activity is a key component of a healthy pregnancy and weight control and focused on walking as a safe and low-impact form of exercise.

As with nutritional advice, a few patients reported that they had received clinic handouts about physical activity but said they had not reviewed the materials with their obstetrician. Others said that they had had general conversations about exercise with their doctors, who encouraged them to continue to be active, especially by walking. Some women reported asking their obstetrician about physical activity. Many women reported that exercise had not been discussed.

### Postpartum weight management

Obstetricians reported that they generally do not directly advise women on postpartum weight loss, either during pregnancy or during the postpartum period.

*I don’t say much at all! And I will say that I’m not actually very diligent about checking their weight at their postpartum [visit] either and comparing it. I think sometimes I do, but it’s certainly not one of the areas I stress.* –Obstetrician, Female

Obstetricians said that postpartum weight loss most often comes up in conversations with patients during discussion of topics that are tangentially related to postpartum weight, such as breastfeeding.

Yet patients discussed several questions and concerns about postpartum weight that they hoped obstetricians would address. Most patients said that they hoped their doctors would give them tips about healthy weight loss and information about the normal timeline for weight loss in the postpartum period, so they could plan and adjust their expectations accordingly. Several women were interested in more specific information about nutrition and appropriate exercise while they were breastfeeding, or advice about when they could safely return to their pre-pregnancy physical activities. Many women expressed general uncertainty about what the post-partum period would be like for their bodies, their moods, and even their relationships with their doctors.

*I don’t even know what kind of support I’m going to get with postpartum-- like I don’t even know if I’m going to see [my obstetrician] or what, so I’m just curious about the kind of the support you get for postpartum weight-loss. You get all of these appointments and everything to talk about when you’re pregnant, and I wonder if there’s a similar sense of having support afterwards.* – Patient, pre-pregnancy BMI 25.1, 14.1 kg (31 pounds) gained at 32.5 weeks

However, not all women desired weight-related postpartum counseling, especially before giving birth. Some said that they feared it might cause counterproductive anticipatory anxiety about the postpartum period, and that they would prefer to address the issue when the time came (though with perhaps a misguided sense of the frequency of seeing the obstetrician postpartum).

*[I don’t want weight loss advice] right now. I'll see [my obstetrician] postpartum frequently enough when we get there. I [think] that if he would have said something to me today about, ‘well, have you thought about postpartum weight?’ I probably would have smacked him.* – Patient, pre-pregnancy BMI 28.8, 15.0 kg (33 pounds) gained at 38 weeks

### Feelings about obstetrician advice

With rare exception, women said they were happy with the obstetric care and counseling they had received. Most said their obstetrician allowed them to raise issues that are of concern, rather than providing information about all possible concerns. Many women appreciated this approach because they feared that too much information about potential problems could lead them to worry unnecessarily, and they trusted that their doctors would alert them to serious problems.

*I feel [my obstetrician is] really approachable if I had any questions that I certainly could ask her…And I think too she's good at not -- she doesn't bring up too many extra concerns. I think that's reassuring to me.* – Patient, pre-pregnancy BMI 23.7, 7.7 kg (17 pounds) gained at 31.5 weeks

A few women said that while they were happy with their obstetrician’s care overall, they would have preferred that their doctor raise issues without waiting for them to ask questions, such as the purpose of GWG, especially since the weight of a newborn constitutes only a fraction of that weight gain. Another woman suggested that obstetricians should perhaps be more forthcoming about the appropriateness of their weight gain and about specific nutrition advice.

*I was just amazed that when I came in for my very first time pregnancy, the doctor doesn’t say, ‘okay, here’s what you can’t eat, here’s what you can’t do, here’s what.’ Six months into the pregnancy I find out you shouldn’t be eating this, or you shouldn’t be doing this, and I thought, ‘well, someone should have said something if that’s really serious for a baby.’* – Patient, pre-pregnancy BMI 18.2, 11.8 kg (26 pounds) gained at 37 weeks

*I think it would have been beneficial once I started hitting closer to that over the normal range or expected range, for someone to say, ‘hey, take a look at what you're doing, you're getting close, let's try to keep your weight down, or in control.'* – Patient, pre-pregnancy BMI 23.7, 22.7 kg (50 pounds) gained at 37 weeks

## Discussion

We found that obstetrician and patient accounts of prenatal communication about GWG diverged in several ways. Compared with their patients’ reports, obstetricians more frequently reported that appropriate weight gain, nutrition, and physical activity were explicitly discussed in prenatal visits. Obstetricians also reported that they tend to offer little weight-related counseling postpartum, while patients expressed anticipatory concerns about postpartum weight loss and a desire for postpartum guidance from their obstetricians.

The obstetricians generally agreed that weight is a medical concern and that pregnancy is an appropriate time to address weight and weight-related behaviors with women; however, they were not always sure about how best to counsel women. This was consistent with another study, in which obstetric care providers said they were “confused about what counseling approach to take, and disagree about how to be effective without offending, stigmatizing, or discouraging patients” [27]. Likewise, patients may be conflicted about weight-related counseling. Patients in our study reported that their obstetricians take a “reactive” approach to counseling, waiting for women to ask questions and raise concerns rather than offering unsolicited advice. This approach to GWG counseling has been previously identified by obstetricians, nurse practitioners, family physicians, and certified nurse midwives in other qualitative studies conducted in the United States [27,29]. Our study supports this finding from the perspective of patients. The patients we interviewed diverged, however, in their feelings about “reactive” counseling practices, with some expressing concern that they may not be receiving all the information they need to have a healthy pregnancy if they do not know what questions to ask providers to draw out appropriate counseling.

Our findings contrast with related work from other countries, including Japan, where researchers found both patients and providers believed minimal GWG was optimal [33], and England, where patients reported that they and their providers were unconcerned about GWG, and providers were not sure how much weight to recommend their patients should gain, or even how much weight they were gaining, since British guidelines do not recommend monitoring GWG [34]. Our study offers evidence of more considered interactions about GWG and a more nuanced approach to weight gain counseling and goal-setting.

Our analysis revealed some discrepancies between obstetricians’ accounts of typical clinical interactions and patients’ accounts. It is possible that when a public health researcher asked them about pregnancy weight gain and how they counsel patients about nutrition and physical activity, physicians may have reported the behavior they knew is best, that is, counseling all patients about healthy behaviors. In a meta-analysis of published research assessing both physician self-report of adherence to evidence-based guidelines and objective measures of adherence, Adams et al. found that the majority of studies shows that physicians self-report that they are much more adherent to medical guidelines in their counseling of patients than they actually are, potentially leading to “gross overestimation of performance” [38]. The authors suspect that physicians are not intentionally misleading researchers, but that they may be subject to desirability bias, which leads them to want to give researchers responses that they deem socially desirable. Alternatively, it is possible that the discrepancies in patient and provider accounts are influenced by patient recall bias. Patients may not fully remember all that their doctors told them earlier in their pregnancies. In particular, women for whom pregnancy weight gain was not a concern may not recall their obstetricians’ comments about weight because other interests trumped those concerns. Other women who indicated that healthy lifestyle advice is common knowledge may have tuned out their physicians’ advice because they presumed nothing novel was being communicated.

### Limitations

This research is subject to several limitations that affect the generalizability of findings. The sample is drawn from a single center and is not representative of all US gravid women or obstetricians. Both obstetrician and patient participants self-selected into the study, raising the possibility that individuals most concerned about GWG were more likely to enroll than individuals who were not concerned with or interested in GWG. Demographic factors may or may not be associated with obstetricians’ weight-related counseling, but future research could target larger and more diverse samples of women and obstetricians to explore whether sample heterogeneity changes findings. In addition, as discussed previously, obstetrician responses may be influenced by desirability bias and patient responses may be affected by recall bias.

## Conclusions

Our study explored patient-provider communication about GWG with diverse obstetricians and patients who were in their third trimester of their first pregnancy. Patient and provider accounts of this communication revealed discrepancies between obstetricians’ perceptions of the counseling they are offering and patients’ perceptions of the counseling they are receiving. This incongruity warrants additional research into the nature and source of divergent patient and provider perspectives on GWG counseling practices. Whatever the cause, our findings suggest that there is a missed opportunity to use prenatal visits as opportunities to discuss healthy eating and exercise during pregnancy, as well as throughout the postpartum period and beyond. Pregnancy brings women into regular contact with the healthcare system, offering opportunities for public health practitioners to interact with a population that is motivated to make healthy lifestyle changes [39]. Additional research testing public health nutrition and physical activity interventions is warranted.

## Abbreviations

BMI: Body mass index; GWG: Gestational weight-gain; IOM: Institute of Medicine.

## Competing interests

The authors declare that they have no competing interests.

## Authors’ contributions

EAD led the study design, collected all data, led data coding and analysis, and drafted the manuscript. EMD contributed to the study design and analysis, and revised the manuscript. KEF contributed to interpretation of results and manuscript revision. All authors read and approved the final manuscript.

## Authors’ information

EAD (PhD) is a postdoctoral research fellow in the Center for Patient Care and Outcomes Research at the Medical College of Wisconsin. Her research interests include women’s health, nutrition and physical activity, and the promotion of healthy lifestyles.

EMD (PhD) is an assistant professor in the Department of Family and Community Medicine at the Medical College of Wisconsin. Her research interests include qualitative methods, community-based participatory research, and chronic disease prevention.

KEF (PhD) is an assistant professor in the Department of Medicine and the Center for Patient Care and Outcomes Research at the Medical College of Wisconsin. Her research interests include the design and analysis of patient-reported outcome measures and patient decision-making.

## Pre-publication history

The pre-publication history for this paper can be accessed here:

http://www.biomedcentral.com/1471-2393/13/231/prepub
